# Assessment of Saliva Specimens' Reliability for COVID-19 Surveillance

**DOI:** 10.3389/fpubh.2022.840996

**Published:** 2022-04-04

**Authors:** Biancamaria Pierri, Maria Tafuro, Maria Concetta Cuomo, Denise Di Concilio, Lucia Vassallo, Andrea Pierri, Amedeo Ferro, Giuseppe Rofrano, Alfonso Gallo, Antonio Di Stasio, Andrea Mancusi, Lydia Galdi, Annachiara Coppola, Carlo Buonerba, Luigi Atripaldi, Pellegrino Cerino

**Affiliations:** ^1^Centro di Referenza Nazionale per l'analisi e studio di correlazione tra ambiente, animale e uomo, Istituto Zooprofilattico Sperimentale del Mezzogiorno, Naples, Italy; ^2^Department of Food Microbiology, Istituto Zooprofilattico Sperimentale del Mezzogiorno, Naples, Italy; ^3^Cotugno Hospital, Azienda Ospedaliera di Rilievo Nazionale Ospedali dei Colli, Naples, Italy

**Keywords:** SARS-CoV-2, saliva, nasopharyngeal swab, real-time RT-PCR, COVID-19 screening

## Abstract

The aim of the present study is to assess saliva as a reliable specimen for severe acute respiratory syndrome coronavirus 2 (SARS-CoV-2) detection by real-time reverse transcription-PCR (RT-PCR), especially in community mass screening programs. The performance analysis considered 1,221 total samples [nasopharyngeal (NP) swabs and corresponding saliva], tested by means of a reference diagnostic real-time RT-PCR assay. Conflicting results were further investigated with a second, more sensitive, reference assay. Analysis of agreement showed a good concordance (95.82%), with a k coefficient value of.74 (*p* < 0.001); moreover, a follow-up analysis revealed the presence of viral gene targets in saliva samples at the time point the corresponding NP swabs turned negative. Data obtained prove the reliability of this alternative biofluid for SARS-CoV-2 detection in real-time RT-PCR. Considering the role of saliva in the coronavirus disease 2019 (COVID-19) transmission and pathogenesis, and the advantages in the use of salivary diagnostics, the present validation supports the use of saliva as an optimal choice in large-scale population screening and monitoring of the SARS-CoV-2 virus.

## Introduction

Since the first outbreak was reported in Wuhan, China in December 2019, the coronavirus disease 2019 (COVID-19) pandemic has caused more than 195 million confirmed cases and more than 4 million deaths worldwide up to July 29, 2021.^1^

The growing worldwide demand for severe acute respiratory syndrome coronavirus 2 (SARS-CoV-2) molecular tests has created significant challenges for clinical and public health laboratories and, according to the WHO recommendations, there is a continuous and critical need for diagnostic testing which is sustainable, practical, and scalable (1).

Real-time reverse transcription-PCR (RT-PCR) represents the current gold standard for SARS-CoV-2 diagnosis, providing a sensitive and specific method to detect SARS-CoV-2 (2).

The molecular method is aimed at detecting the RNA of the virus in biological specimens as respiratory samples, including the following: nasopharyngeal (NP) swabs, oropharyngeal (OP) swabs, and bronchial aspirate which are the most commonly analyzed (2). Despite NP/OP swabs being the principal collection method for identification of SARS-CoV-2 (3), it represents an invasive process that can cause discomfort to the patient and a high risk of contagion for healthcare workers (4). By contrast, saliva is emerging as a good supplemental or alternative to NP/OP swabs ^1^ forCOVID-19 diagnosis and monitoring (5), especially in those less developed countries with limited resources (6, 7). It represents a sensitive biofluid for screening of asymptomatic or pre-symptomatic SARS-CoV-2 infections and for viral load monitoring (5). Actually, salivary droplets represent the main source of the human-to-human transmission of SARS-CoV-2 infection (8). The SARS-CoV-2 virus infects humans through the respiratory tract or conjunctival mucosa and has a preferential tropism to human cells expressing cellular receptors for angiotensin-converting enzyme 2 (ACE2) (9). The expression of ACE2 receptor is higher on the epithelial cells of the oral mucosa and the minor salivary glands, suggesting them as a repository of the virus (4, 5, 10).

Some studies have evaluated the accuracy and feasibility of saliva for SARS-CoV-2 detection (1) and the stability of saliva samples collected and transported without specialized collection devices or media (11). It has also been demonstrated as a good concordance with paired NP/OP swabs in SARS-CoV-2 detection (3, 12). However, the role of saliva in COVID-19 diagnosis is not limited to a qualitative detection of the virus, but it could also provide information about the clinical evolution of the disease (8), together with other biological markers (13).

The aim of the present study is the assessment of saliva as a reliable tool to detect SARS-CoV-2, and we propose it as an alternative biological specimen for COVID-19 large-scale screening and monitoring programs.

## Materials and Methods

### Sampling

From each subject, the NP swab and the saliva sample were collected simultaneously. For the collection of saliva samples, participants were asked to produce saliva in their mouth for a few minutes and gently spit about 1.5 ml into a sterile nuclease-free 50 ml collection container. After the collection, a 2:3 ratio of a phosphate buffer saline (PBS, Sigma-Aldrich, USA) solution at a pH of 7.4 (Vacuette REF 456162, Greiner Bio-One International GmbH, Austria) was immediately added to the tubes in order to dilute samples and allow long-term storage. NP was collected by means standard tube with a virological transport medium (Vacuette REF 456162, Greiner Bio-One International GmbH, Austria).

### RNA Extraction

Through an automated nucleic acid platform (Maelstrom 9600, TANBead, Taiwan), RNA was extracted with a magnetic bead-based protocol, using a TANBead Nucleic Acid Extraction Kit (TANBead, Taiwan). According to the manufacturer's instructions, 300 μl was the input material for each sample. The RNA was finally eluted in 80 μl of elution buffer in the dedicated plate provided with the kit.

### Real-Time RT-PCR

As reference diagnostic assay, the real-time PCR was performed using 5 μl of the extracted RNA according to the Allplex 2019-nCoV Assay (Seegene, South Korea) protocol, considering E, RdRp/S, and N as target genes for the detection of SARS-CoV-2. Real-time RT-PCR was set on the CFX-96 Bio-rad instrument (Bio-rad, USA).

A second reference diagnostic assay was performed for further investigation (AbAnalitica, Italy). In brief, 10 μl of the extracted RNA was used according to Real Quality RQ-2019-nCoV (AbAnalitica, Italy) protocol, considering RdRp and E as target genes for the detection of SARS-CoV-2. Real-time RT-PCR was performed on the Agilent AriaDX instrument (Agilent, USA).

### Data Analysis

Each result was validated after the positive and the negative controls have been examined. A re-test was performed if the Internal Control (IC) showed no value or Ct ≥ 40. Results were interpreted according to the manufacturer's instructions which are as follows: Allplex 2019-nCoV Assay (Seegene, South Korea) considered the sample as “SARS-CoV-2 not detected” in the absence of amplification for all targets; “SARS-CoV-2 detected” if amplification of all targets or two of them was achieved; the same results was given also if one gene between RdRp/S or N was amplified. The amplification of the only E gene gave inconclusive results. The declared limit of detection (LOD) for this analytical assay was 100 g.c./5 μl reaction.

Discordant samples were tested by means of a second reference diagnostic assay, Real Quality RQ-2019-nCoV (AbAnalitica, Italy). A sample was considered positive if amplification of both RdRp and E gene was achieved, negative in the absence of amplification for both targets, and inconclusive when only one of the targets was amplified. The declared LOD for this analytical assay was a 3 g.c./10 μl reaction.

### Assessment of Performance Characteristics

Sensitivity (Se, proportion of positive samples correctly identified as positive), specificity (Sp, proportion of negative samples correctly identified as negative), and accuracy (Ac, the proportion of correct assessments over the total number of assessments; also named in the text as “Concordance”) with corresponding 95% CI were calculated for the reference diagnostic assays tested on saliva samples, considering the exclusion of samples (n.51) still conflictual after the in-depth analysis by means of the second reference diagnostic assay. The k coefficient (14) was considered to estimate the agreement between the saliva real-time RT-PCR and NP swabs real-time RT-PCR results. Statistical calculations were done using R statistical software (R Core Team, Austria) (15).

## Results

Table 1 summarizes data obtained on 1,221 total NP swabs and saliva, tested by means of the real-time RT-PCR reference diagnostic assays (detection of E, RdRp/S, and N genes). On the total of samples analyzed SARS-CoV-2 was detected in 134 NP swabs, while 1,081 NP swabs resulted as SARS-CoV-2 negative. Among the results, NP swabs gave inconclusive results (only E gene amplification). Concerning saliva samples, SARS-CoV-2 was detected in 87 of them and not detected in 1,127, whereas seven salivas resulted as inconclusive.

**Table 1:** Summary of study results with the first reference diagnostic assay (Allplex 2019-nCoV—Seegene).

				**Biological Specimens**		
	**E**	**RdRp/S**	**N**	**NP swabs (no. of samples)**	**Saliva (no. of samples)**	**Results**
Reference assay	+	+	+	134	87	SARS-CoV-2 detected
	+	+	-			
	+	-	+			
	-	+	+			
	-	+	-			
	-	-	+			
	+	-	-	6	7	Inconclusive
	-	-	-	1,081	1,127	SARS-CoV-2 not detected

**Figure 1:**
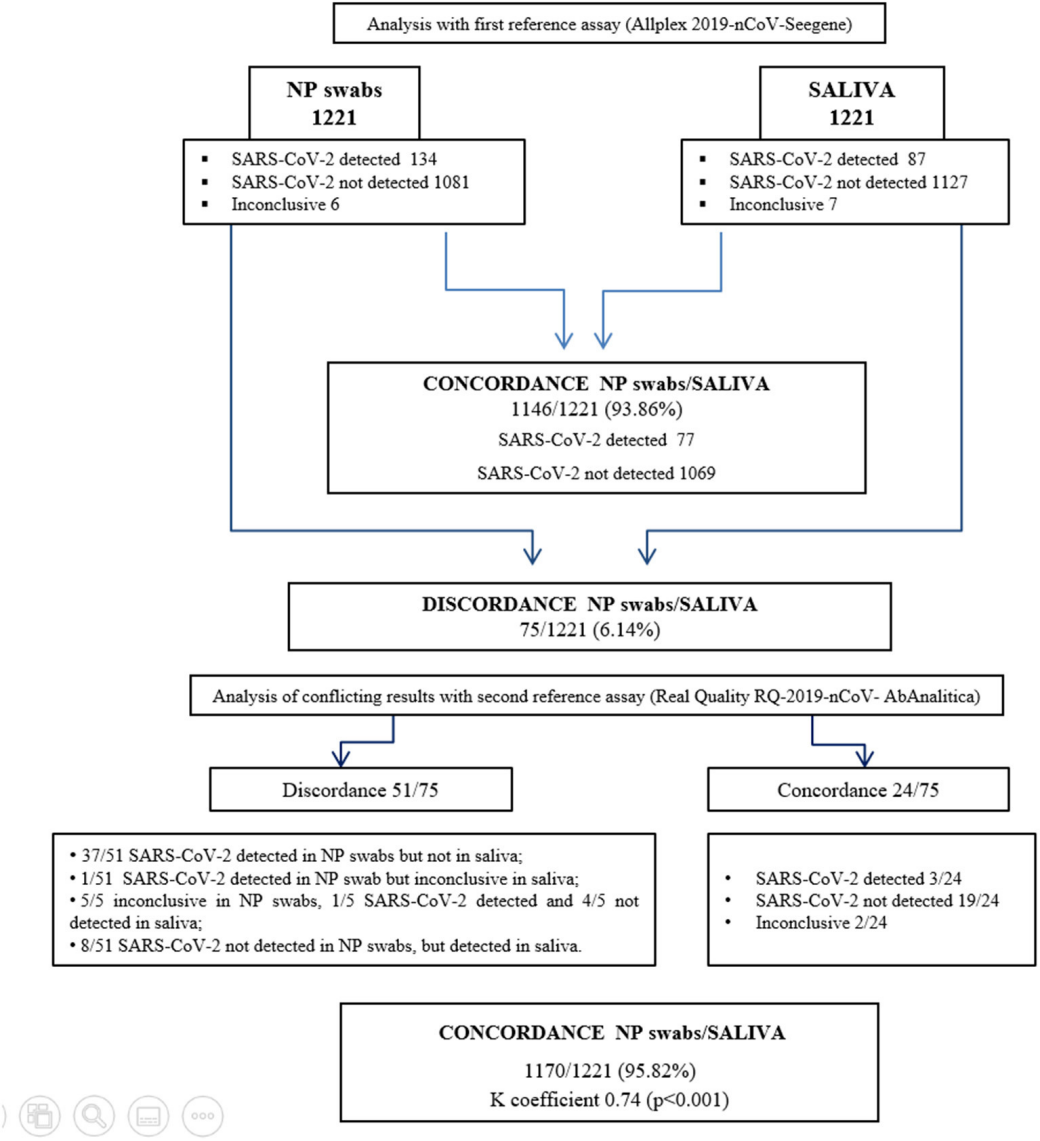
Analytical workflow of the study.

Figure 1 shows the analytical workflow. Agreement of results between the reference diagnostic assay tested on the two types of biological specimen was achieved on 1,146 samples (77 positive and 1,069 negative). Thus, comparing the NP swabs and saliva analysis, the percentage of concordance (that also corresponded to the accuracy of the method) was 93.86% (1,146/1,221), while the percentage of discordance was 6.14% (75/1,221).

Discordance was observed on 75 samples, among which five samples SARS-CoV-2 was detected with Ct > 34 in NP swabs, while an exclusive amplification of the E gene gave inconclusive results in the corresponding saliva samples. Furthermore, six samples resulted inconclusive in the NP swabs and negative in the corresponding saliva, while two negative NP swabs resulted as inconclusive saliva. For 10 samples where SARS-CoV-2 was not detected in NP swabs, it was instead detected in the corresponding saliva. Finally, for 52 samples where the virus was detected with high Ct values (24 NP swabs with three genes amplification, Ct ≥ 26; 14 NP swabs with 2 genes amplification, Ct ≥ 35; 14 NP swabs with one gene amplification, Ct ≥ 35), the corresponding saliva was negative. Discordant results were further investigated with a second reference diagnostic test (detection of E and RdRp genes), gaining agreement between the two types of specimens in 24/75 samples, moving the concordance to 95.82% (1,170/1,221) and discordance to 4.18% (51/1,221). The agreement, measured as k coefficient, was 0.74 (95% CI.68–0.81; *p* < 0.001) (Supplementary Figure S1). (Raw data are shown in Supplementary Tables 1, 2).

**Figure 2:**
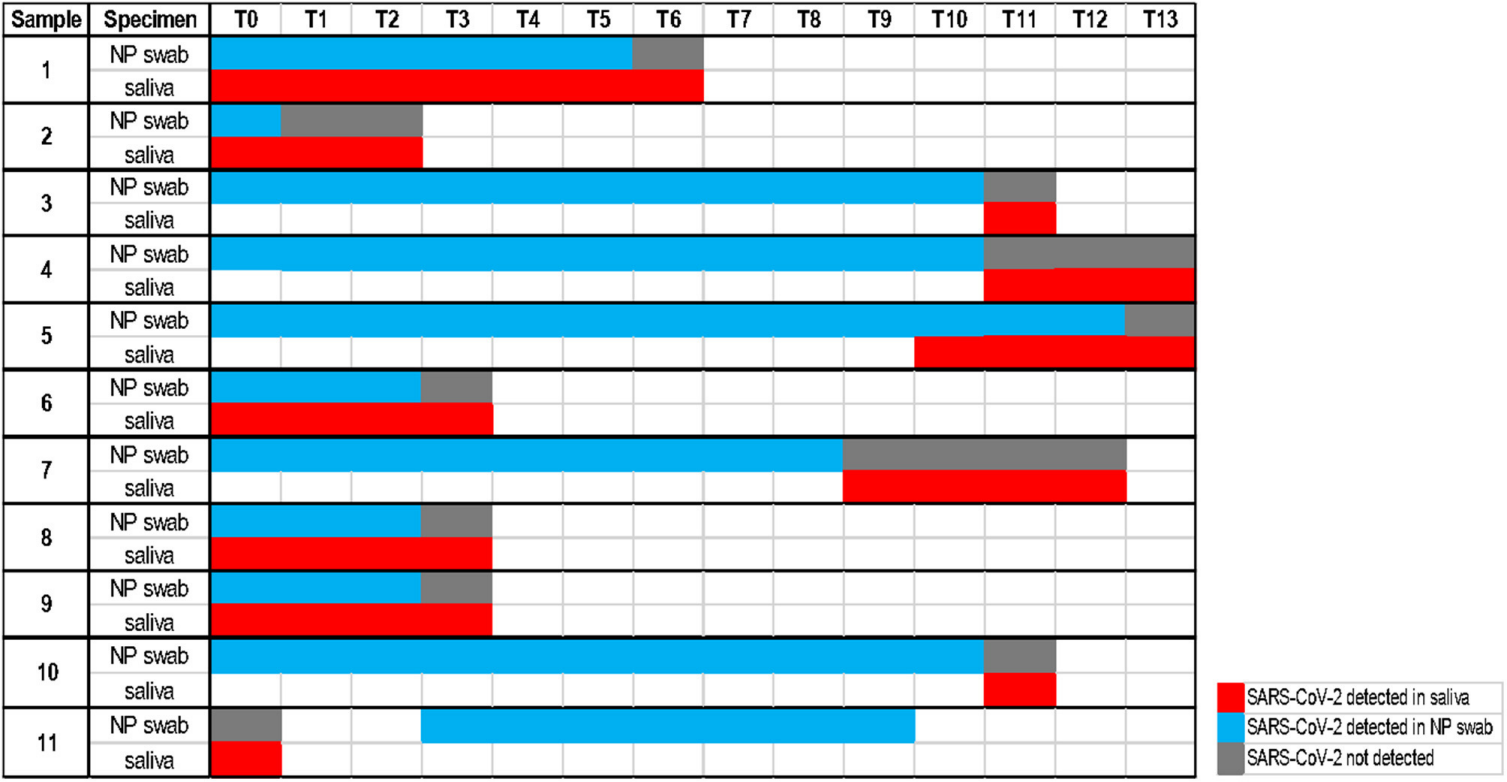
Timeline with a follow-up analysis on 11 discordant cases (each time point considers an interval of one day).

The calculated performance characteristics (95% CI), considering the saliva as an alternative biological specimen, were the following: Se 78.1% (71.6–84.7) and Sp 100% (99.7–100). Among the 51 discordant results, 11 of them were followed in the course of the illness. As shown in Figure 2, for 10 cases, saliva remained positive at the time point the NP swabs turned negative. On the other hand, one case of positive saliva represents instead an early detection of the positivity revealed in the following NP swabs.

## Discussion

Respiratory samples, and particularly NP swabs, tested by means of real-time RT-PCR assays, are currently considered the gold standard specimen for the detection of the SARS-CoV-2 virus (2, 16). Nevertheless, some critical points have emerged in the process of NP swabs collection: patients' discomfort, children's refusal or difficulties, close contact between infected people and healthcare workers, time required for sampling, etc. (4, 8). Since the earliest months of COVID-19 spreading, many authors have explored the use of alternative biological specimens as promising tools for SARS-CoV-2 detection (8, 17). The meta-analysis carried out by Bwire et al. (18) showed the highest positivity rate of SARS-CoV-2 detection in lower respiratory tract samples, as the bronchoalveolar lavage fluid (BLF) and the sputum, as well as in rectal swabs. The most commonly and widely used NP, less invasive if compared to BLF, instead revealed a moderate positivity detection rate. Similar results were obtained by Wang et al. (19), who detected the virus from multiple sites, showing a lower positivity rate from NP swabs (63%), compared to BLFs (93%) and sputum (72%). Considering, on one hand, the invasiveness of BLF sampling, and on the other, it is well known that dry cough is one of the most common symptoms of the COVID-19, in mild to severe illness conditions (20), and this comes down to a limited chance to produce and collect sputum samples from coughing or phlegm expulsion. By contrast, saliva, produced by drooling or self-collection, is emerging in the SARS-CoV-2 diagnostic scenario as a good alternative of the biological non-invasive specimen (5), already proved as an affordable and rapid matrix for detection of other viruses (21). Comparison between NP swabs and saliva in several studies have underlined the good concordance between the two types of specimens in COVID-19 testing, with saliva diagnostic performances comparable to the current standards (22).

Our investigation showed, on a total of 1,221 NP swabs and corresponding saliva samples tested by means of two reference diagnostic assays in real-time RT-PCR, a concordance of 95.82%, with Se and Sp of 78.1 and 100%, respectively. The k coefficient value confirmed a good agreement of detection between the NP swabs and the saliva samples (k coefficient 0.74, 95% CI.68–0.81), and it was found statistically significant (*p* < 0.001). These results are consistent with Pasomsub et al. (23) observations, who demonstrated in saliva high sensitivity and comparable performance to the current standard of nasopharyngeal and throat swab, revealing, on a total of 200 samples, an analysis of agreement of 97.5% (K coefficient 0.85, 95% CI.72-0.98; *p* < 0.001). Likewise, Guclu et al. (6) observed, on a total of 64 oro-nasopharyngeal swabs and saliva samples, a substantial agreement with a k coefficient of 0.74 (*p* < 0.001).

Moreover, our follow-up analysis on 11 discordant cases (Figure 2) showed the persistence of the detectable virus in 10 saliva samples at the time point the corresponding NP swabs become negative. This could be easily understood considering that the SARS-CoV-2 virus could infect humans through the respiratory apparatus and may migrate in saliva droplets from the lower or upper respiratory tract, from the blood into gingival crevicular fluid, or by salivary glands infection (24). The high expression of ACE2 receptors on the epithelial cells of the salivary gland and of the oral mucosa, reported for SARS-CoV in rhesus macaques (25), suggests the potential active role of the oral cavity, of the salivary glands and then of the saliva in the pathogenesis and transmission of COVID-19 (8, 10). SARS-CoV-2 may persist in saliva droplets making this biological fluid an optimal candidate for virus detection and infectious monitoring. In addition, one case of our follow-up analysis allowed early detection of the virus, confirmed by the positivity of the following NP swab. This result is consistent with observations of Liu et al. (25) on positive saliva produced by infected salivary glands in early infection of SARS-CoV.

The limit of the present study emerges in the observation of a percentage of discordant results (4.18%). Even if these conflicting data were obtained on samples with high Ct values and with target concentrations close to the analytical LOD of the reference diagnostic assays, it is symptomatic of a need for standardization parameters in saliva sampling. This means the definition of a minimum amount of saliva representative of the viral presence (also in the condition of low viral load), or the use of passive drool devices for sampling, rather than the patients' self-collection procedure. Although the saliva diagnostic sensitivity appeared lower than NP swabs (26), adopting saliva as a first-line test in community mass screening programs could present many advantages. Firstly, the process of saliva sampling, by drooling or by self-collection, avoids the risk of healthcare workers' exposure and the patients' discomfort given its non-invasiveness. It also reduces the time required for NP swabs collection by specialized personnel, representing a suitable alternative in countries with low-resource possibilities (26).

In view of a daily life restart, saliva appears to be an optimal choice for large population-level screenings (i.e., schools), as rapid-collection, non-invasive, specific specimens for SARS-CoV-2 monitoring. There is a wide interest in the use of saliva as a reference biofluid for the early diagnosis of several diseases (not limited to infective ones) (27). Indeed, given the evolution of diagnostic technologies, it can serve as a reliable tool for mass population screening, allowing the detection of biomarkers by means of proteomics, transcriptomics, metabolomics, microRNAs, and microbiomics approaches (28). In the “salivaomics” definition of Wong, it is implied the translational and clinical vision of salivary diagnostics: the characteristics of accessibility of this type of specimen and the connection to systemic diseases give to saliva the possibility to be the optimal choice for the advancement of point-of-care medicine, as it is already happening in liquid biopsy research field (29). Thus, salivary diagnostics can contribute to health and disease surveillance and to personalized medicine advance, with a considerable utility not only in the context of the COVID-19 pandemic.

## Data Availability Statement

The raw data supporting the conclusions of this article will be made available by the authors, without undue reservation.

## Ethics Statement

The studies involving human participants were reviewed and approved by AOU Federico II Ethics Committee. The patients/participants provided their written informed consent to participate in this study.

## Author Contributions

BP and PC: conceptualized the study. BP, MT, MC, DC, LV, and PC: methodology and data analysis. BP, MT, MC, DC, LV, AP, AC, and LG: data curation. AP: programmed and managed the software. AP, AF, GR, AG, and AM: formal analysis. BP and AS: project administration. BP, MT, MC, DC, and LV: wrote the original draft. BP, CB, LA, and PC: supervised and verified the underlying data. All authors contributed to the article and approved the submitted version.

## Conflict of Interest

The authors declare that the research was conducted in the absence of any commercial or financial relationships that could be construed as a potential conflict of interest.

## Publisher's Note

All claims expressed in this article are solely those of the authors and do not necessarily represent those of their affiliated organizations, or those of the publisher, the editors and the reviewers. Any product that may be evaluated in this article, or claim that may be made by its manufacturer, is not guaranteed or endorsed by the publisher.

## Footnotes

^1^ www.who.int
