# Supplementary figures and images for: Assessment of Saliva Specimens' Reliability for COVID-19 Surveillance
Source: Front Public Health. 2022 Apr 4;10:840996. doi: 10.3389/fpubh.2022.840996 (PMC9015071; doi:10.3389/fpubh.2022.840996)

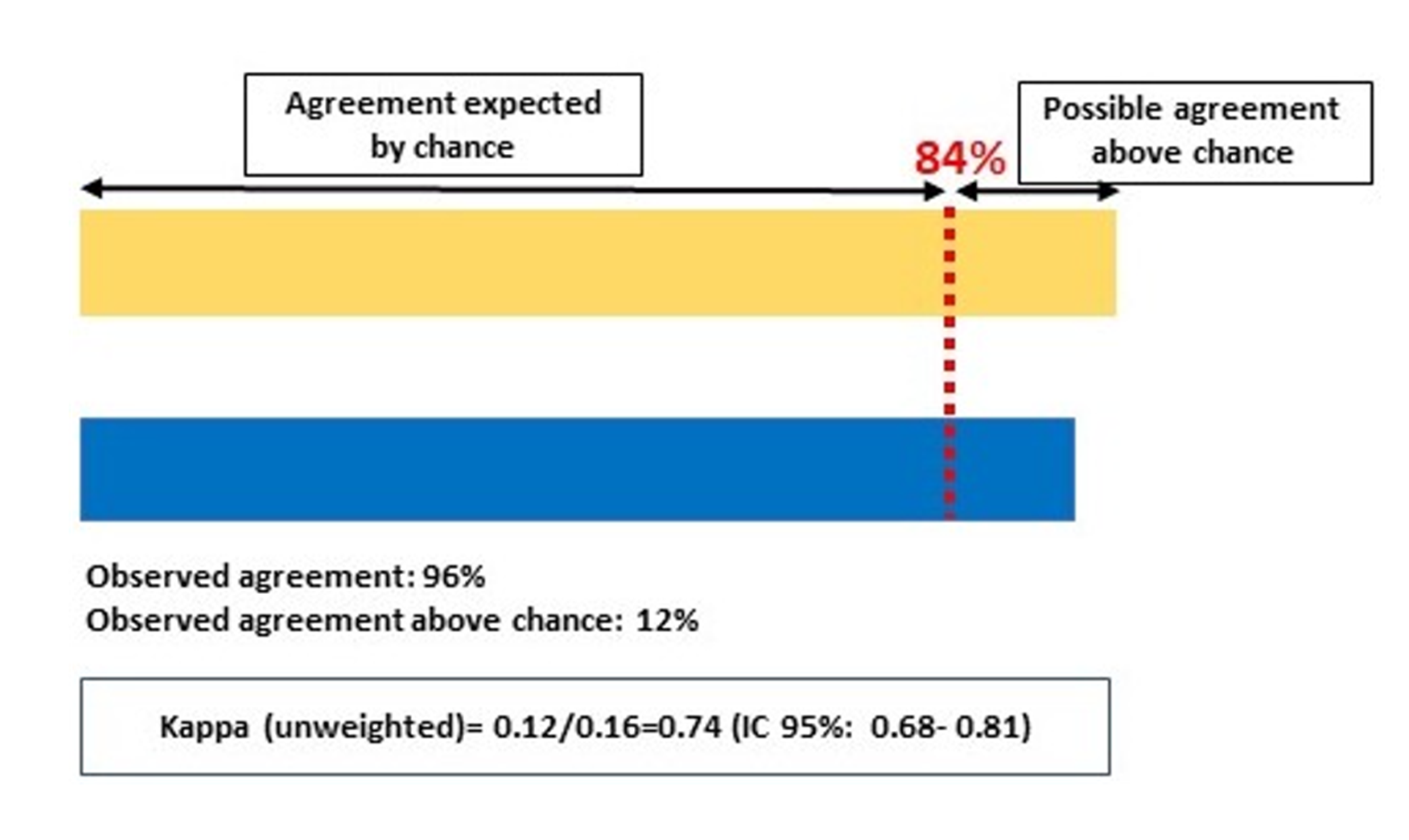

Supplement: Supplementary file 3 [file Image_1.TIF]
